# Feeding regulates sex pheromone attraction and courtship in *Drosophila* females

**DOI:** 10.1038/srep13132

**Published:** 2015-08-10

**Authors:** Sébastien Lebreton, Federica Trona, Felipe Borrero-Echeverry, Florian Bilz, Veit Grabe, Paul G. Becher, Mikael A. Carlsson, Dick R. Nässel, Bill S. Hansson, Silke Sachse, Peter Witzgall

**Affiliations:** 1 Swedish University of Agricultural Sciences, Department of Plant Protection Biology, Division of Chemical Ecology, Alnarp, Sweden; 2 Department of Evolutionary Neuroethology, Max Planck Institute for Chemical Ecology, Jena, Germany; 3 Colombian Corporation of Agricultural Research Research CORPOICA, Biological Control Laboratory, Las Palmas 240142, Colombia; 4 Department of Zoology, Stockholm University, Stockholm, Sweden

## Abstract

In *Drosophila melanogaster*, gender-specific behavioural responses to the male-produced sex pheromone *cis*-vaccenyl acetate (cVA) rely on sexually dimorphic, third-order neural circuits. We show that nutritional state in female flies modulates cVA perception in first-order olfactory neurons. Starvation increases, and feeding reduces attraction to food odour, in both sexes. Adding cVA to food odour, however, maintains attraction in fed females, while it has no effect in males. Upregulation of sensitivity and behavioural responsiveness to cVA in fed females is paralleled by a strong increase in receptivity to male courtship. Functional imaging of the antennal lobe (AL), the olfactory centre in the insect brain, shows that olfactory input to DA1 and VM2 glomeruli is also modulated by starvation. Knocking down insulin receptors in neurons converging onto the DA1 glomerulus suggests that insulin-signalling partly controls pheromone perception in the AL, and adjusts cVA attraction according to nutritional state and sexual receptivity in *Drosophila* females.

*“The preservation of animals is dependent on their ability to find food and to propagate, and for these practical purposes it is the very sense of smell that counts”* (Titus Lucretius Carus, De Rerum Natura).

Food intake is an essential component of sexual reproduction in animals, who accordingly need to harmonize the search for food and mates, and the sensory cues that encode them. Insects use sex pheromones for premating communication. Sex pheromones are not perceived alone, but in blends with habitat and food signals, which enhance their behavioral activity^1^,^2^. The neural circuitry underlying the integration of these two types of chemosensory cues is a target for sexual and natural selection, and accordingly salient for reproductive isolation and speciation^3^,^4^.

Fruit flies *Drosophila melanogaster* gather and mate on decaying and fermenting fruit^5^,^6^. Yeast growing on fruit serves as an essential part of the adult and larval diet and flies are accordingly attracted to fermentation metabolites^7^,^8^,^9^. During mating, males release the volatile sex pheromone *cis*-vaccenyl acetate (cVA), which increases female receptivity^10^ and functions as an aggregation pheromone, since it enhances male and female attraction to food odour^11^,^12^. Odours emanating from food also act as aphrodisiacs by themselves and promote male courtship^13^,^14^, which further emphasizes the interconnection between pheromone and food odour communication in *Drosophila*.

Female receptivity to male courtship is regulated by *doublesex* neurons, which are responsive to cVA^15^. Male courtship, on the other hand, is largely determined by the *fruitless* (*fru*) transcription factor^16^,^17^,^18^. Females and males perceive olfactory signals via shared first-order olfactory neurons, while gender-specific differences in response to sex pheromone^15^,^19^,^20^,^21^ and food odour^13^ become apparent in third-order olfactory neurons, some of which broadly respond to both types of odour^21^. It remains, however, unknown how food odours modulate the response to pheromone.

Insects and other animals adjust their sexual behaviour according to mating and nutritional state; the sensory and behavioural responses to sex and food signals are therefore under coincident modulation^22^,^23^,^24^,^25^. Acute perception of cVA via Or67d (and the DA1 glomerulus) enhances female sexual receptivity in *Drosophila*^10^, whereas chronic exposure and perception via Or65a (DL3) mediates an aversive effect of cVA in both sexes^26^,^27^. Interestingly, short neuropeptide F (sNPF), which is regulated by insulin according to nutritional state and modulates food attraction, is strongly expressed in these cVA-responsive DA1 and DL3 glomeruli^28^,^29^.

This led to the hypothesis that perception of pheromone and food signals is under concurrent modulation in *Drosophila*. We show for the first time that nutritional state has an effect on female attraction to blends of male sex pheromone cVA and food odour, and that first-order olfactory circuits in the AL contribute to this gender-specific behavioural modulation.

## Results

### Food intake has a sex-specific effect on pheromone attraction

**Figure 1:**
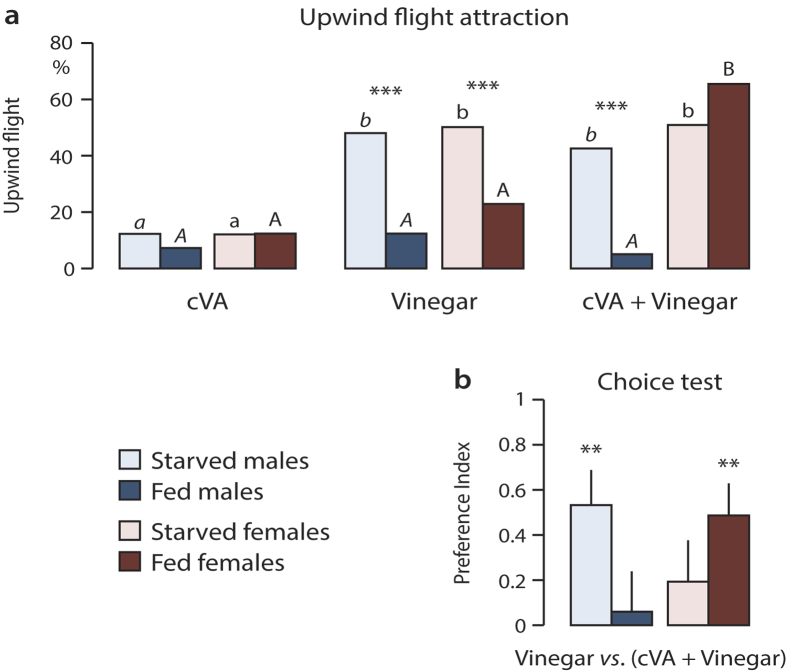
Nutritional state and cVA attraction. Attraction of starved and fed unmated *D. melanogaster* males and females (n = 40) to cVA, in a flight tunnel (**a**) and a y-tube olfactometer (**b**) bioassay. Wind tunnel: upwind flight attraction to single odour sources (letters show significant differences between insects of same sex and feeding state, in response to different odour sources; asterisks show significant differences between starved and fed flies of the same sex to the same stimulus; GLM, Wald test, ***p < 0.001). Olfactometer: choice test between a blend of cVA and vinegar vs. vinegar alone. Asterisks indicate significant attraction (mean ± SEM, Wilcoxon test, **p < 0.01).

Male sex pheromone cVA enhanced upwind flight attraction of fed females to vinegar. Both starved and fed females were attracted, while significantly fewer fed than starved males responded to this mixture of cVA and vinegar. Vinegar alone attracted fewer fed than starved flies, of both sexes. Flies were only weakly attracted to cVA alone (Fig. 1a). In a dual choice test, fed but not starved females showed a preference for the blend of cVA and vinegar, over vinegar alone. In comparison, fed and starved males showed an opposite response (Fig. 1b).

**Figure 2 f2:**
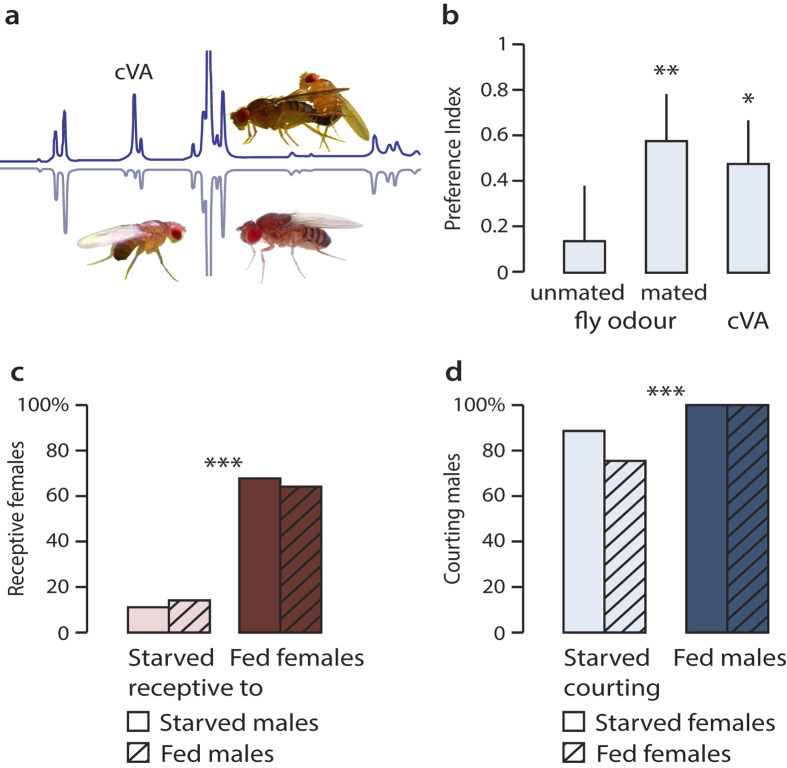
Behavioural context: effect of cVA release on attraction and effect of starvation on courtship. (**a**) Chromatograms showing volatiles released by mating (top) and non-mating flies (bottom trace). Release of cVA increased from 8.1 ± 0.3 in non-mating flies (n = 8) to 43.4 ± 3.0 pg/min/fly in mating flies (n = 6) (Mann-Whitney test, V = 48, p < 0.001). (**b**) Male attraction towards a blend of vinegar and pheromone (volatiles collected from mating flies, non-mating flies, or synthetic cVA), compared to vinegar alone in a y-tube olfactometer. Males were attracted by volatiles from mating flies (n = 20), and not to volatiles from single flies (n = 22). Synthetic cVA equivalent to the amount released by mating flies (n = 25) induced significant attraction (Wilcoxon’s signed rank test; *p < 0.05, **p < 0.01). (**c**) Sexual receptivity of fed and starved females courted by either starved or fed males. (**d**) Effect of starvation of male courtship behaviour, towards either fed or starved females. Asterisks (**c,d**) show a significant effect of starvation (GLM, ***p < 0.001; n = 30). Photos by S. Lebreton.

Males transfer cVA to females during mating^11^,^27^,^30^,^31^ and the combination of cVA and food odour signals aggregation and mating sites. The behavioural effect of increased cVA release during mating, and of starvation on courtship is shown in Fig. 2. Mating flies release significantly more cVA than unmated flies (Fig. 2a). Starved males responded more strongly to volatiles released by mating flies, or to corresponding amounts of synthetic cVA, than to volatiles released by unmated flies (Fig. 2b).

Responsiveness of fed female flies to cVA and vinegar (Fig. 1) may accordingly reflect sexual receptivity and attraction to mating sites. This was corroborated by testing the effect of starvation on mating behaviour: sexual receptivity of females depended significantly on nutritional state, disregarding the state of courting males (Fig. 2c). The effect of starvation and feeding on male mating activity was less pronounced (Fig. 2d).

### Starvation differentially affects vinegar and pheromone perception in the ALs of females

**Figure 3:**
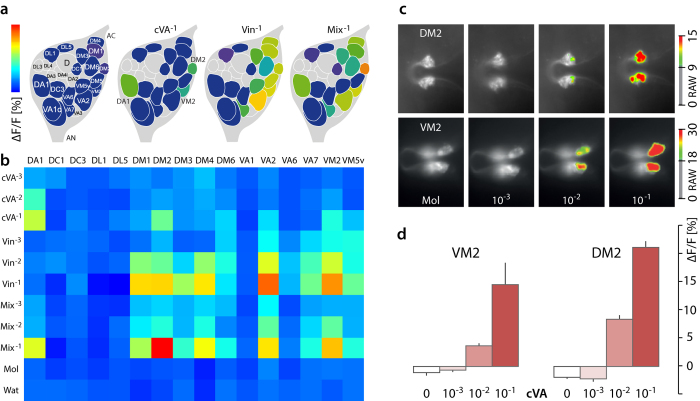
Glomerular activation patterns in the AL of fed females, in response to cVA and vinegar. (**a**) Schematic dorsal view of a *D. melanogaster* antennal lobe (AL). Coloured glomeruli (n = 17) were reliably identified (66), remaining glomeruli are greyed out. Colours show the median normalized calcium activity (ΔF/F [%]) in response to controls and odor applications, according to the colour bar on the left. Antennal nerve (AN), antennal commissure (AC). (**b**) Heat odour map showing the calcium imaging response of 16 glomeruli to cVA, vinegar (Vin) and a blend of both (Mix), in 3 dilutions, 10^−3^ to 10^−1^ and the solvents, mineral oil (Mol) and water. Each data point shows the median glomerular response from ten fed females, responses were normalized to the highest response in each fly. Colours show the median normalized calcium activity (ΔF/F [%] (see colour bar above). (**c**) Calcium imaging response in 4-d-old males to three dilutions of cVA (10-3 to 10-1) and solvent (Mol). Two fly lines, Or22a-GAL4 and Or43b-GAL4 were used for imaging the DM2 (top) and VM2 (bottom) glomeruli. Median normalized calcium activity (ΔF/F [%]), according to the colour bar on the right. (**d**) Median normalized calcium activity (ΔF/F [%]; n = 10) in response to cVA, in DM2 and VM2 glomeruli (see **c**).

**Figure 4:**
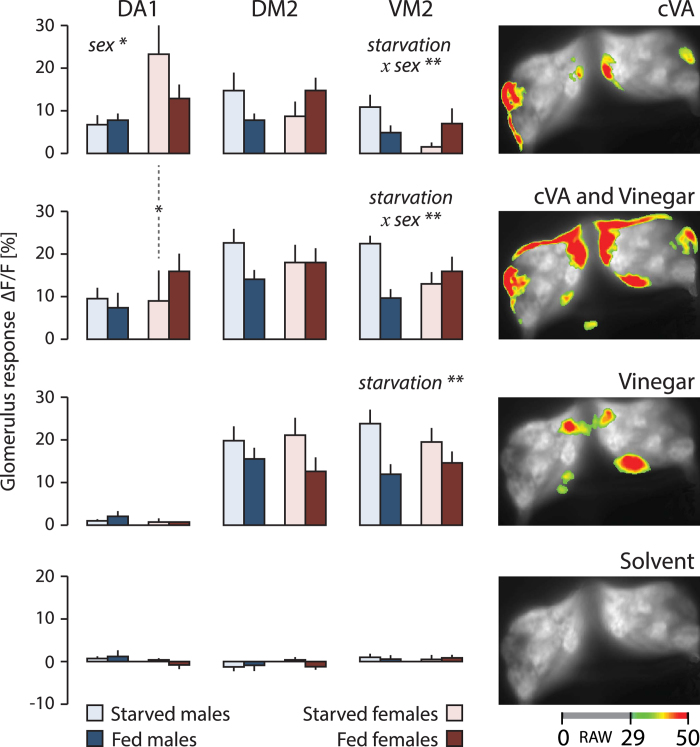
Activation of three cVA-responsive glomeruli (DA1, DM2, VM2) in the AL of starved and fed flies in response to cVA and vinegar. Effect of starvation on calcium responses evoked by cVA, vinegar and a blend of cVA and vinegar in three glomeruli (DA1, DM2 and VM2) responding consistently to cVA (see Fig. 3). DM2 and VM2, but not DA1, responded to vinegar; solvent (mineral oil) did not elicit a significant response. Median normalized calcium activity (ΔF/F [%]), according to the colour bar at the bottom. Males and females, starved or fed (n = 8) were tested, stimuli were presented in a 10^−1^ dilution. Effect of sex, starvation and the interaction of these two factors (starvation × sex) on the response elicited by each stimulus in each glomerulus were tested using a two-way ANOVA (*p < 0.05, **p < 0.01). The response to cVA alone and the blend of cVA and vinegar in starved females was compared with a Wilcoxon test (V = 34, p = 0.023).

We next analysed the effect of starvation on the AL response to cVA, vinegar and to a blend of cVA and vinegar, using functional imaging of olfactory sensory neurons (OSNs), by driving GCaMP expression under control of the Orco-GAL4 line. The DA1 glomerulus responded specifically to cVA, and not to vinegar alone (Figs 3a,b, and 4). Responses in DA1 were recorded at dilutions of 10^−2^ and 10^−1^ (Fig. 3a). In addition, stimulation with the highest cVA dose (10^−1^), elicited consistent responses in the DM2 and VM2 glomeruli (Figs 3a,b, and 4). This was confirmed by testing cVA in the Or22a-GAL4 and Or43b-GAL4 lines (Fig. 3c,d). Ten glomeruli (DM1, DM2, DM3, DM4, DM5, DM6, VA2, VA7, VM2 and VM5v) responded to vinegar, at dilutions between 10^−3^ and 10^−1^ (Fig. 3b).

The effect of sex and starvation on the activity of cVA, vinegar and their blend in DA1 (responding to cVA) and in DM2 and VM2 (responding to both vinegar and cVA) is shown in Fig. 4. cVA elicited a stronger response in DA1 in females than in males, and its response was not significantly affected by starvation in either sex. Interestingly, adding vinegar to cVA significantly decreased the DA1 response in starved females, but had no effect in fed females (Fig. 4).

Nutritional state had a sexually dimorphic effect in the VM2 glomerulus in response to cVA. Interestingly, the same response pattern was observed with a blend of cVA and vinegar, but not with vinegar alone. This suggests that cVA counteracts the effect of starvation on vinegar perception in females, but not in males (Fig. 4).

### Insulin signalling in specific OSNs is required to induce cVA attraction in fed females

In *Drosophila*, sensitivity towards food odour is increased by starvation and reduced by feeding^29^,^32^,^33^,^34^. Our results show that the response of fed males to a blend of vinegar and cVA is also reduced. In contrast, in females, feeding does not decrease flight attraction to a cVA/vinegar blend. Fed, but not starved, females even prefer this blend over vinegar alone. Therefore, we further investigated the physiological response to cVA in females.

During starvation sNPF signalling in specific OSNs facilitates synaptic transmission and therefore increases food perception at the postsynaptic level in the AL^29^. After feeding insulin-like peptides (ILPs) are released from insulin-producing cells (IPCs) in the brain^35^ and activate the insulin receptor (InR) in OSNs, which in turn suppresses expression of the sNPF receptor and thus decreases food odour sensitivity^29^.

**Figure 5:**
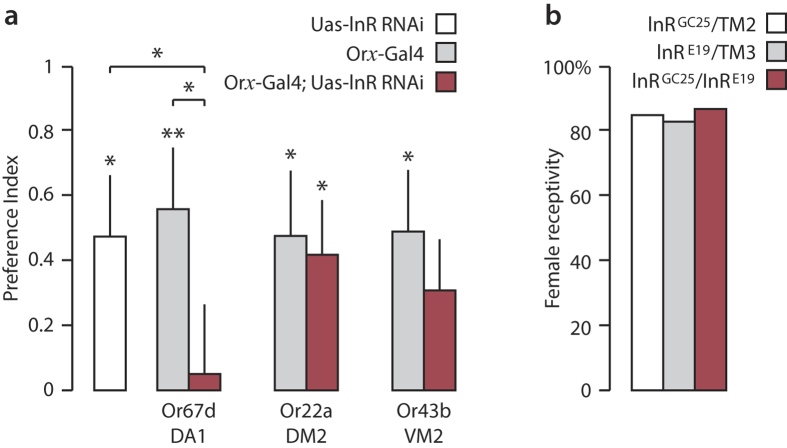
Effect of insulin signalling on female attraction to cVA and sexual receptivity. (**a**) Effect of knocking down InR in three OSN subpopulations projecting to DA1 (Or67d-GAL4), DM2 (Or22a-GAL4) and VM2 (Or43b-GAL4) glomeruli on cVA attraction in fed females (asterisks above bars show significant attraction to cVA; mean + SEM, Wilcoxon test, *p < 0.05, **p < 0.01; asterisks between bars show significantly different preference indices between InR knocked-down flies and control lines, GLM, *p < 0.05; n = 20 to 32). (**b**) Sexual receptivity of InR mutant (InR^GC25^/InR^E19^; n = 24) fed females compared to corresponding controls (InR^GC25^/TM2 (n = 28) and InR^E19^/TM3 (χ^2^-test, p = 0.88; n = 35).

We tested whether the insulin-signalling pathway is also involved in regulating cVA attraction in females in response to feeding. To this purpose we knocked down insulin signalling, in OSNs projecting to specific glomeruli, using InR RNAi in fed females (Fig. 5a). We selected the DA1 glomerulus, which is known to be involved in cVA detection^36^, and the DM2 and VM2 glomeruli, which both responded to cVA and vinegar (Figs 3 and 4). All control lines (uas-InR RNAi, Or67d-Gal4, Or22a-Gal4 and Or43b-Gal4) showed a significant preference for the blend of cVA and vinegar. Knocking down insulin signalling in the cVA-specific glomerulus DA1 almost entirely suppressed the preference for cVA in fed females (Fig. 5a). This suggests that insulin signalling in Or67d-expressing OSNs is necessary to trigger cVA attraction in fed females. When InR was knocked down in VM2, preference for cVA was no longer significant. However, the behavior of these flies did not significantly differ from their control parental lines and a role of VM2 in the regulation of cVA attraction could thus not be confirmed. Knocking down InR in DM2 had no effect.

Finally, we tested whether the effect of starvation on female sexual receptivity to male courtship depends on insulin signalling (Fig. 5b). Towards this goal, we used a temperature sensitive mutant of InR^37^. These flies exhibit an InR mutant phenotype when the temperature is raised to 25 °C. Flies were reared at 17 °C to avoid developmental defects due the lack of InR during larval development and were kept at 25 °C after adult emergence. Sexual receptivity of fed females was not affected by the lack of InR (Fig. 5b). Therefore, unlike cVA attraction, the insulin signalling pathway has no effect on female receptivity.

## Discussion

### Integration of food and sex signals

**Figure 6:**
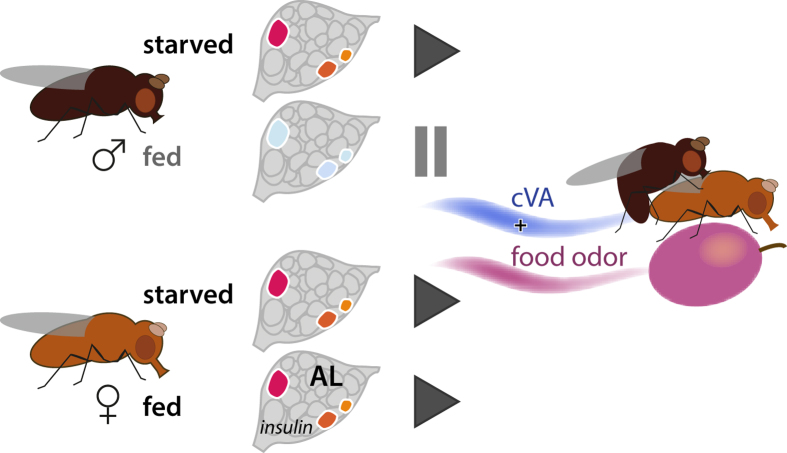
Graphical abstract. Starved insects, females and males, are attracted to food odour. Fed females, which are receptive to male courtship, but not fed males, are attracted to blends of cVA and food odour. Insulin signalling in first-order olfactory neurons in the antennal lobe (AL), in DA1 and VM2 glomeruli, contributes to this behavioural reaction.

*Drosophila* males and females meet on ripe fruit where they feed, mate and oviposit^6^,^38^. Accordingly, they perceive food olfactory cues and pheromones as an ensemble. That environmental and social cues cannot be dissociated in natural habitats is reflected by the behavioural and chemical ecology of the fly. Grosjean *et al.*^13^ established how food odours enhance the sexual behaviour of *Drosophila* males. Projection neurons downstream of sensory neurons dedicated to pheromone and food odours converge in the pheromone processing region of the lateral horn, to promote male courtship behaviour. We here show that females and males use a first-order olfactory pathway for the integration of male-produced sex pheromone cVA and food signals, and that the female behavioural response to sex and food odours is modulated by its nutritional state, which also influences sexual receptivity (Fig. 6).

The male-produced sex pheromone cVA functions to increase female receptivity to male courtship^10^,^39^. Our behavioural studies of a blend of cVA and food odour vs. food odour alone show behavioural synergism and a response modulation in fed females, and demonstrate that the olfactory pathways responding to these signals are interconnected. Starved females prioritize the search for food, cVA has no effect on their upwind flight response (Fig. 1a) and their odour preference in a choice test (Fig. 1b). Fed females, on the other hand, which are sexually receptive (Fig. 2), showed a clear response to the blend of cVA and food odour (Fig. 1). Fed males, in comparison, showed little activity in response to olfactory stimuli (Fig. 1). Unlike females, males preferred cVA only when starved, supporting the idea that starvation increases odour sensitivity in males, disregarding the nature of the stimulus.

Adult *Drosophila* females require nutrient intake for reproductive functions, including oogenesis^40^,^41^. An association between nutritional state and reproductive behaviour is a well-conserved feature in many other animals^42^,^43^ and even in mammals, a decrease in sexual receptivity is accompanied by a loss of preference for social odours signals^44^.

### Sex-specific modulation of the cVA pathway in the AL

A sexually dimorphic behavioural response to cVA, i.e. increased female receptivity to male courtship vs. male-male aggression and courtship inhibition, relies on sexually dimorphic third-order neurons^15^,^17^,^19^,^20^,^21^. Food-related odour, by itself, enhances male courtship behaviour through activation of sexually dimorphic courtship circuitry^13^.

The modulation of cVA perception in starved vs. fed females shown here effects first-order olfactory neurons in the AL (Figs 3 and 4). cVA stimulates the DA1 glomerulus^10^. In addition, it elicits a response in two isomorphic glomeruli, DM2 and VM2, which also respond to vinegar odour (Figs 3 and 4). The response pattern in VM2 to cVA, as well as the behavioral response to a blend of cVA and food odours are starvation-dependent and gender-specific (Figs 1 and 4). It remains to be determined how olfactory input modulation and behavioral response modulation are interconnected.

### Regulation of odour-mediated attraction by insulin

The global metabolic cue insulin and local signalling with short neuropeptide F (sNPF) have been shown to interact in the AL to regulate the attraction response to food cues according to nutritional state. Following feeding, insulin (via activation of InR) inhibits the expression of sNPF receptors in DM1 OSNs and therefore decreases sensitivity to food odours by reducing synaptic transmission^29^. Our results confirm that DM1, DM2 and DM4 glomeruli, which respond to starvation^29^, are activated by vinegar odour (Fig. 3b). Disruption of insulin signalling in DA1, on the other hand, induces a loss of the preference for cVA in fed females (Fig. 5a). This suggests that insulin acts on the female olfactory system to regulate pheromone attraction.

Insulin is a key regulator of insect development, metabolism and behaviour^29^,^37^,^45^,^46^,^47^,^48^. The role of insulin in regulating *Drosophila* sexual behaviour remains, nonetheless, controversial. Although insulin regulates female remating, it does not affect sexual receptivity in unmated females^45^,^49^, which we confirm by using a temperature-sensitive mutant of InR (Fig. 5b). This suggests that the nutritional state regulates both pheromone perception and sexual receptivity in females through two distinct mechanisms. Insulin signalling is required, at least, in the DA1 glomerus to induce pheromone attraction (Fig. 5a) and in the DM1 glomerulus to reduce food attraction^29^ in fed *Drosophila* females. The mechanisms by which the same hormonal pathway can both up- and downregulate sensitivity to different odours are yet unknown. A combination of excitatory and inhibitory local interneurons or projection neurons, receiving differential OSN input, may underlie such a bimodal response.

Another scenario pertains to the participation of sugar receptors in feeding-induced olfactory response modulation. Sugar receptors function to sense external, as well as internal sugars in the hemolymph^50^ and very recently, it has further been shown that antennal neurons, expressing Gr64b together with Orco, coincidently project to DA1 and VM2^51^. This finding will certainly stimulate future work on the physiological mechanisms regulating sexual behaviour as a function of nutritional state in *Drosophila*.

## Conclusion

*Drosophila* courtship is a classical paradigm for studying the neural logic of innate behaviour. Research emphasis has been placed on the male-produced sex pheromone cVA and the neural circuits encoding sex-specific behavioural responses^15^,^21^. The DA1 glomerulus is known to contribute to cVA attraction^52^. We show that cVA activates also the sexually isomorphic DM2 and VM2 glomeruli, which respond to vinegar, and that perception of cVA and food odour interacts in these glomeruli, in a gender-specific fashion (Figs 3, 4, 5). It follows that investigations of physiological and behavioural responses to cVA should take habitat or food odours into account, since in nature, the flies perceive social and environmental signals as an ensemble.

The behavioural response to olfactory stimuli is not a constant, but is modulated, following mating or feeding, to match physiological internal states^23^,^53^,^54^,^55^. Our behavioural tests (Figs 1, [Fig f2] and 2) show that the olfactory attraction to food odour and sex pheromone is modulated according to nutritional state and sexual receptivity.

## Materials and Methods

### Insects

The Dalby strain^56^ of the fruit fly *Drosophila melanogaster* was used as a wild-type strain. For functional imaging experiments the following transgenic lines were used: Orco-GAL4; Or22a-GAL4; Or43b-GAL4^57^,^58^; UAS-GCaMP3^59^. In order to manipulate the activity of InR in OSNs, a line expressing an InR RNAi (UAS-InR RNAi) was used^60^. This transgene was expressed in subpopulations of OSNs using specific GAL4 drivers (Or67d-GAL4, Or22-GAL4 and Or43b-GAL4)^61^. A global temperature sensitive InR mutant was obtained as previously described^37^: two transgenic lines (InR^[E19]^/TM2 and InR^GC25^/TM3) were crossed and the resulting trans-heterozygous InR^[E19]^/ InR^GC25^ was a temperature sensitive mutant of InR; InR^[E19]^/TM3 and InR^GC25^/TM2 were used as controls.

Flies were reared on a standard sugar-yeast-cornmeal medium diet under a 12:12 h L:D photoperiod. Newly emerged flies were anesthetized with CO_2_ and separated by sex under a microscope. Flies of the same sex were then kept in 30-ml plastic tubes with fresh diet (fed flies) or with a humidified piece of cotton wool (starved flies). Wild-type flies were kept at room temperature while transgenic flies were kept at 25 °C. InR mutants show a mutant phenotype when the temperature is raised to 25 °C. In order to avoid any defect of the lack of InR during larval development, these flies were reared at 17 °C and kept at 25 °C after adult emergence. Wild-type flies were starved for 3 d while transgenic flies were starved for 1–2 d before tests. Fly lines were obtained from the Bloomington Drosophila Stock Center (IN, USA) and the Vienna RNAi Stock Center (Austria).

### Behavioural analysis

Upwind flight attraction was observed in a wind tunnel^62^ made of glass, with a 30 × 30 × 100 cm flight section. An airstream of 0.25 m/s was produced by a fan (Fischbach GmbH, Neunkirchen, Germany), which was filtered and homogenized by an array of four activated charcoal cylinders (14.5 cm ø, 32.5 cm long; Camfil, Trosa, Sweden). The tunnel was lit diffusely from above, at 13 lux, temperature ranged from 20 °C to 22 °C, relative humidity from 42% to 48%. Odours were delivered from a piezoelectric sprayer^63^, driven by a microinjection pump (CMA Microdialysis AB, Solna, Sweden). 40 insects were flown to each test odour. Flies were scored for flying upwind from a release tube at the end of the tunnel over 80 cm towards the odour source, which was concealed by a wire mesh.

A y-tube olfactometer with two branches (2 cm × 30 cm glass tubes) and an air-stream of 0.25 m/s was used. 25-ml glass vials were vertically connected with a ground glass fitting at the inlet of each branch; these vials were either empty or filled with 8 ml of vinegar, to provide a vinegar odour background^12^. In addition, cVA and hexane, respectively, were released at a rate of 10 μl/min into the olfactometer branches from a piezoelectric sprayer (see above).

Fed and starved *D. melanogaster* males and females were tested (n = 40). Single 3-d-old flies were introduced at the entrance of the Y-tube and the time spent in each branch was recorded. Tests lasted 5 min. An attraction index (AI) was calculated as follows: AI = (time spent in branch with cVA – time spent in control branch)/(time spent in cVA branch + time spent in the control branch). The AI is 1, when flies remain in the stimulus branch during the entire test; AI is –1, when flies remain in the control branch; AI is 0, when test flies spend the same amount of time in both branches. Only flies that became activated when exposed to the odour stimulus were taken into account.

Female sexual receptivity was tested with single fly pairs. One randomly selected female (fed or starved) and one random male (fed or starved) were placed in round dishes (45 mm diameter × 30 mm high). All combinations were tested (n = 30 fed males/fed females, n = 20 fed males/starved females, n = 40 starved males/fed females, n = 20 starved males/starved females). InR mutant and control females were individually mated with a random wild-type starved male; males displaying courtship and females mating within 1 h were recorded.

### Odor collection

Fifteen to 16 flies (unmated females and males or copulating flies) were placed in a glass vial with a narrow capillary-like outlet^64^. Charcoal-filtered air (0.9 l/min) was blown with an aquarium pump into the vial. Chemicals released by the flies were collected on the glass surface. After 75 min, flies were removed and vials were rinsed three times with 100 μl of hexane. Two types of extracts were prepared: one with chemicals produced by copulating flies, the other with a mix of chemicals produced by virgin flies of both sexes (with a female/male ratio of 1:1).

### Chemical analysis

Heptadecenyl acetate (100 ng) was added to 50 μl of the two previously described extracts as an internal standard (n = 6 for mating flies, n = 8 for non-mating flies). These extract were then analysed on a gas chromatograph coupled with a mass spectrometer (GC-MS; 6890 GC and 5975 MS, Agilent technologies Inc., Santa Clara, CA, USA); 2 μl of the extracts were injected into a HP-5MS silica capillary column (30 m × 0.25 mm × 0.25 μm film thickness; Agilent Inc.) which was temperature-programmed from 30 °C to 225 °C at 8 °C/min. The amount of cVA in mating and non-mating fly headspace collections was quantified by peak integration of diagnostic fragments for cVA (m/z = 250) and internal standard (m/z = 83). cVA was identified according to its mass spectrum and retention time.

### Optical imaging

Flies were prepared for optical imaging as described by Strutz *et al.*^65^. We used a Till Photonic imaging system with an upright Olympus microscope (BX51WI) and a 20x Olympus objective (XLUM Plan FL 20x/0.95W). A Polychrome V provided light excitation (475 nm), which was then filtered (excitation: SP500, dicroic: DCLP490, emission LP515). The emitted light was captured by a CCD camera (Sensicam QE, PCO AG) with a symmetrical binning of 2 (0.625 × 0.625 μm/pixel). For each measurement, a series of 40 frames was taken at 4 Hz. Odor was applied after 1.5 s, during frames 6–14 (2 s).

cVA (Pherobank, Wageningen, The Netherlands) was diluted in mineral oil (Carl Roth GmbH, Germany) from 10^−1^ to 10^−3^; white wine vinegar (Mezzocorana, Italy) was diluted from 10^−1^ to 10^−3^ in distilled water. 6 μl of these dilutions were pipetteted onto filter paper (~1 cm^2^, Whatman) and placed in Pasteur pipettes. For tests of 2-component blends, two filter papers were placed into the same pipette. Filter papers with solvent alone were used as blanks. Filter papers were prepared ca. 30 min before tests. A stimulus controller (Stimulus Controller CS-55, Syntech) was used for odor application. Continuous airflow (1 l/min), monitored by a flowmeter (0.4–5 LPM Air, Cole-Parmer) was directed through an acrylic glass tube to the fly’s antennae. Odor stimuli were injected into this air stream.

Data from *in vivo* recordings were processed by custom-written IDL software (ITT Visual Information Solutions). All recordings were manually corrected for movement. For the calculation of relative fluorescence changes (ΔF/F), the fluorescence background was subtracted from the averaged values of frames 0 to 6 of each measurement. The false color-coded fluorescent changes in raw data images were calculated by subtracting frame 7 from 12.

A 3-D map of the fly AL^66^ served to link the active area to individual glomeruli. All experimental flies contained the calcium dependent fluorescent sensor G-CaMP3^59^ together with a promoter GAL4 insertion to direct expression of the calcium sensor to specific neuron populations. Stimulus-evoked fluorescence in these flies arises from the population of labelled neurons that are sensitive to the specific odour. We tested the physiological responses in input neurons, i.e. the axonal terminals of OSNs in the AL. Mass labelling of OSNs was achieved by using the transgenic line Orco-GAL4 that drives expression in at least 60% of all OSNs^58^.

### Statistical analysis

Wind tunnel attraction data were analysed using a Generalized Linear Model (GLM) with a Bernoulli binomial distribution. Post-hoc Wald pairwise comparison tests identified differences between treatments. Attraction index (AI) values were compared to a theoretical value of 0 (no attraction), using a Wilcoxon’s signed rank test. To test the effect of starvation, sex, vinegar, and the interaction of these factors on cVA attraction, we used a Mixed-Effects Linear Model with the pheromone treatment as a random effect. cVA preferences between transgenic lines were compared using a GLM with a quasibinomial family followed by a multiple comparison analysis with a fdr correction method (multcomp package). The effect of male and female starvation state on mating behaviour was analysed using a GLM with a binomial error distribution. A χ^2^-test was used for male courtship and female receptivity in InR mutants. Optical imaging data were analysed using a two-way ANOVA. The amounts of cVA released by mating and non-mating flies were compared using a non-parametric Mann-Whitney test. Statistical analyses were computed with R (R 2.1.1, R Development Core Team, Free Software Foundation Boston, MA, USA).

## Additional Information

**How to cite this article**: Lebreton, S. *et al.* Feeding regulates sex pheromone attraction and courtship in *Drosophila* females. *Sci. Rep.* **5**, 13132; doi: 10.1038/srep13132 (2015).
